# Performance Evaluation of 18 Generative AI Models (ChatGPT, Gemini, Claude, and Perplexity) in 2024 Japanese Pharmacist Licensing Examination: Comparative Study

**DOI:** 10.2196/76925

**Published:** 2025-09-18

**Authors:** Hiroyasu Sato, Katsuhiko Ogasawara, Hidehiko Sakurai

**Affiliations:** 1 Department of Pharmacy, Abashiri-Kosei General Hospital, Abashiri, Japan; 2 Graduate School of Pharmacy, Hokkaido University of Science, 7-Jo 15-4-1 Maeda, Teine, Sapporo, 006-8585, Japan, 81 11 681 2161, 81 11 681 3622; 3 Graduate School of Health Sciences, Hokkaido University, Sapporo, Japan; 4 Graduate School of Engineering, Muroran Institute of Technology, Muroran, Japan

**Keywords:** generative AI, artificial intelligence, ChatGPT, Gemini, pharmacist, National License Examination

## Abstract

**Background:**

Generative artificial intelligence (AI) has shown rapid advancements and increasing applications in various domains, including health care. Previous studies have evaluated AI performance on medical license examinations, primarily focusing on ChatGPT. However, the availability of new online chat-based large language models (OC-LLMs) and their potential utility in pharmacy licensing examinations remain underexplored. Considering that pharmacists require a broad range of expertise in physics, chemistry, biology, and pharmacology, verifying the knowledge base and problem-solving abilities of these new models in Japanese pharmacy examinations is necessary.

**Objective:**

This study aimed to assess the performance of 18 OC-LLMs released in 2024 in the 107th Japanese National License Examination for Pharmacists (JNLEP). Specifically, the study compared their accuracy and identified areas of improvement relative to earlier models.

**Methods:**

The 107th JNLEP, comprising 345 questions in Japanese, was used as a benchmark. Each OC-LLM was prompted by the original text-based questions, and images were uploaded where permitted. No additional prompt engineering or English translation was performed. For questions that included diagrams or chemical structures, the models incapable of image input were considered incorrect. The model outputs were compared with publicly available correct answers. The overall accuracy rates were calculated based on subject area (pharmacology and chemistry) and question type (text-only, diagram-based, calculation, and chemical structure). Fleiss’ κ was used to measure answer consistency among the top-performing models.

**Results:**

Four flagship models—ChatGPT o1, Gemini 2.0 Flash, Claude 3.5 Sonnet (new), and Perplexity Pro—achieved 80% accuracy, surpassing the official passing threshold and average examinee score. A significant improvement in the overall accuracy was observed between the early and the latest 2024 models. Marked improvements were noted in text-only and diagram-based questions compared with those of earlier versions. However, the accuracy of chemistry-related and chemical structure questions remains relatively low. Fleiss’ κ among the 4 flagship models was 0.334, which suggests moderate consistency but highlights variability in more complex questions.

**Conclusions:**

OC-LLMs have substantially improved their capacity to handle Japanese pharmacists’ examination content, with several newer models achieving accuracy rates of >80%. Despite these advancements, even the best-performing models exhibit an error rate exceeding 10%, underscoring the ongoing need for careful human oversight in clinical settings. Overall, the 107th JNLEP will serve as a valuable benchmark for current and future generative AI evaluations in pharmacy licensing examinations.

## Introduction

Generative artificial intelligence (AI) development has been remarkable in recent years and has been adopted in many fields, including education and health care. There have been reports that generative AI has been used to summarize clinical texts [1-4] and has been introduced into clinical practice [5,6]. Furthermore, the potential benefits of generative AI in medical education have been explored [7-10], and its usefulness has been demonstrated in the writing and publishing of medical research [11].

In the United States, generative AI has been implemented in 86% of health care organizations [12]. Moreover, approximately 40% of health care professionals use generative AI at their workplaces at least once a week [13]. Correspondingly, online chat-based large language models (OC-LLM) have attracted the attention of many users because of their ease of use. In health care, the use of OC-LLMs can have serious consequences if their performance is inadequate. Therefore, verifying the knowledge base and problem-solving capabilities of OC-LLMs in health care settings is essential.

A wealth of information is available on the web in the medical and health care domains, and OC-LLMs acquire a substantial amount of knowledge during pretraining. In addition to general medical knowledge, pharmacists must have expertise in fields, such as physics and chemistry, which differ from those required by other health care professionals. However, few studies have evaluated the performance of OC-LLMs in pharmacies. The performance of ChatGPT (GPT-3.5 and GPT-4V models) in the Japanese National License Examination for Pharmacists (JNLEP) was evaluated by Sato and Ogasawara [14]. Since then, numerous new OC-LLM services and models have been released in 2024. However, the performance of these newly released models in the field of pharmacy has not been sufficiently evaluated. Furthermore, it was hypothesized that each OC-LLM service (ie, ChatGPT, Gemini, Claude, and Perplexity) has distinct strengths and limitations.

Accordingly, the purpose of this study is to evaluate the performance of various OC-LLMs introduced in 2024 in the field of pharmacy using the JNLEP and to assess performance improvements in the latest models.

## Methods

### Services and Models

The following 18 OC-LLMs, all available as of 2024, were evaluated (Table 1): ChatGPT (7 models), Gemini (4 models), Claude (5 models), and Perplexity (2 models). Claude 3.5 Sonnet (new) was renamed as Claude 3.5 Sonnet in June 2024, and as of January 2025, these models are the most commonly used OC-LLMs. Microsoft Copilot, one of the most popular OC-LLMs [15], was excluded because its underlying engine, GPT-4 (released in 2023), was evaluated in a previous study as the model used in ChatGPT and is mainly used for tasks other than digital browser-based dialogues. Although Copilot has continued to improve in terms of functionality and performance, the details of its current model and update history remain undisclosed. Consequently, this was excluded from the 2024 OC-LLM performance evaluation in this study.

**Table 1.** Characteristics, release dates, and evaluation dates of the OC-LLM^a^ services and models used in this study.

Service and model	Deprecated or active^b^	Uploadable image	Release date^c^	Evaluation date^d^
ChatGPT				
GPT-3.5	Deprecated	No	November 2022	May 2024
GPT-4	Active	Yes	September 2023	November 2023
GPT-4o mini	Active	No	July 2024	July 2024
GPT-4o	Active	Yes	May 2024	May 2024
o1 mini	Active	No	September 2024	October 2024
o1 preview	Deprecated	No	September 2024	September 2024
o1	Active	Yes	December 2024	December 2024
Gemini				
1.0 Pro	Deprecated	Yes	February 2024	May 2024
1.5 Pro	Active	Yes	May 2024	May 2024
1.5 Flash	Active	Yes	May 2024	August 2024
2.0 Flash Experimental	Active	Yes	December 2024	December 2024
Claude				
3 Haiku	Deprecated	Yes	March 2024	June 2024
3 Sonnet	Deprecated	Yes	March 2024	May 2024
3 Opus	Active	Yes	March 2024	June 2024
3.5 Sonnet	Active	Yes	June 2024	June 2024
3.5 Sonnet (new)	Active	Yes	October 2024	November 2024
Perplexity				
Standard	Active	No	June 2024	November 2024
Pro	Active	Yes	June 2024	December 2024

a OC-LLM: online chat-based large language model.

b Status of each model, whether deprecated or active as of January 1, 2025.

c Release data of each used model in Japan.

d Performance evaluation date of each model used in this study.

### Japanese National License Examination for Pharmacists

This study used 345 questions from the 107th JNLEP held in February 2022. This dataset is the same as that used by Sato and Ogasawara [14]. The questions in the 107th JNLEP are organized into the following 9 subject categories: physics, chemistry, biology, hygiene, pharmacology, pharmaceuticals, pathophysiology, regulations, and practice. All questions were presented in a multiple-choice format, requiring the selection of 1 or 2 correct answers from the 5 options. The passing criteria for the 107th JNLEP included an overall accuracy of at least 62.9% along with 2 additional conditions. The details of the 107th JNLEP were extensively covered by Sato and Ogasawara [14].

### Data Measurement

All OC-LLMs, except for ChatGPT GPT-4, were evaluated for their performance from May to December 2024. The data outcomes for ChatGPT GPT-4 were collected from a preliminary study conducted in November 2023 [14]. For ChatGPT GPT-3.5, a preliminary test was conducted in February 2023. However, a new evaluation was conducted in May 2024 to assess the potential performance improvements in the same model.

For all models, the complete set of questions from the 107th JNLEP was input in Japanese in order of the question numbers. Although response performance can be improved through prompt engineering [16-18], no prompts were used in this study.

For questions that included diagrams or charts, the questions and options were input as text, whereas the diagram or chart portion was input as an image. Some early models could not process the diagrams (Table 1); therefore, these questions were omitted and marked as incorrect.

### Data Analysis

The output from each OC-LLM was compared with publicly available correct answers [19] to determine whether the responses were correct or incorrect. An incorrect answer (ie, hallucinations) was defined as a response in which the selected answer differed from the published correct answer, the specified number of answers was not selected, or no answer was provided. Even when the correct option number could not be explicitly identified in the output by the OC-LLMs, the response was considered correct if the selected content matched the correct answer choice. The accuracy of each model was evaluated based on the total number of subjects and question types (text only, including diagrams, calculations, chemical structures, and graphs). Question-type classification was subjectively determined by the researcher based on the content of the questions. Questions with diagrams were also counted as those containing graphs or chemical structures. The calculated questions included text-only and diagram-based questions.

To assess improvements in model accuracy, statistical comparisons were performed between the 3 model outputs (ChatGPT GPT-4, Gemini 1.0 Pro, and Claude 3 Sonnet) released in early 2024 and those of the latest 4 flagship models (ChatGPT o1, Gemini 2.0 Flash Experimental, Claude 3.5 Sonnet [new], and Perplexity Pro).

Answer consistency was used to validate whether the tasks in which the generative AI model excelled or struggled showed similar trends across models based on the highest accuracy model of each service.

### Statistical Analysis

A generalized linear mixed model (GLMM) was used to evaluate the accuracy improvements. The correctness of the responses to each question was set as the dependent variable. The model type (early or latest), question type (text-based or diagram-based), and their interactions were specified as fixed effects. Models and questions were included as random effects. Fleiss’ κ [20] was used to assess the consistency of responses. All statistical analyses were performed using R (version 4.4.2; R Foundation for Statistical Computing).

## Results

### Performance Statistics of AI Models

The performances of 18 generative AI models from 2024 in the pharmaceutical field were evaluated (Table 2). The performance of the 4 flagship models (ChatGPT o1, Gemini 2.0 Flash Experimental, Claude 3.5 Sonnet [new], and Perplexity Pro) was over 80%, which was markedly higher than that of the passing criteria. When reassessed, ChatGPT GPT-3.5 recorded an overall accuracy of 38.8% (134/345), indicating no marked progress from its former performance of 35.4% (122/345), showing no substantial improvement.

**Table 2.** Overall accuracy of each OC-LLM^a^ on the 107th JNLEP^b^.

Service and model	Correct answers^c^	Overall accuracy	Passing criteria^d^
ChatGPT			
GPT-3.5	134	0.388	Failed
GPT-4o mini	215	0.623	Failed
o1 mini	234	0.678	Passed
GPT-4^e^	250	0.724	Passed
o1 preview	272	0.788	Passed
GPT-4o	294	0.852	Passed
o1	299	0.866	Passed
Gemini			
1.0 Pro	171	0.495	Failed
1.5 Flash	242	0.701	Passed
1.5 Pro	246	0.713	Passed
2.0 Flash	288	0.834	Passed
Claude			
3 Sonnet	194	0.562	Failed
3 Haiku	213	0.617	Failed
3 Opus	260	0.753	Passed
3.5 Sonnet	293	0.849	Passed
3.5 Sonnet (new)	297	0.860	Passed
Perplexity			
Standard	228	0.660	Passed
Pro	301	0.872	Passed

a OC-LLM: online chat-based large language.

b JNLEP: Japanese National License Examination for Pharmacists.

c Number of correct answers out of all 345 questions in the 107th JNLEP.

d Overall accuracy>62.9%.

e GPT-4 results were obtained from Sato and Ogasawara [14].

**Figure 1.**
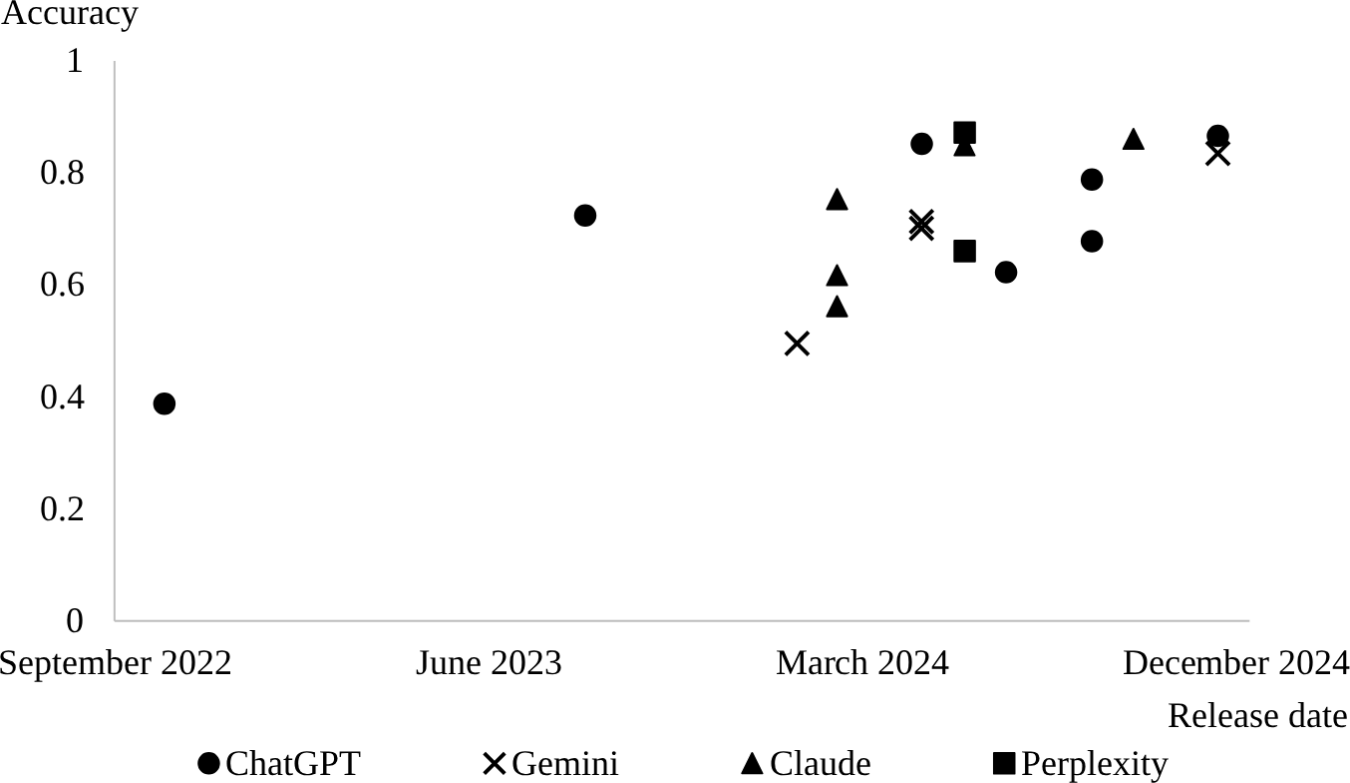
Relationship between release date and accuracy in each online chat-based large language model service.

For all services, the model enhancements were confirmed to result in increased accuracy. All the models released after September 2024, regardless of whether they were light, medium, or high, met the qualification criteria (Figure 1). All GPT-4 results were obtained from Sato and Ogasawara [14]. The raw data of each model’s item-by-item correctness are presented in Multimedia Appendix 1.

### Performance of AI Models According to Subject and Question Type

By subject, pathophysiology and pharmacology showed high accuracy for all models except for the ChatGPT GPT-3.5 model. In the most recent models, the accuracy in pharmaceuticals and biology improved substantially, whereas in physics and chemistry, only minor improvements were observed (Table 3). In the 4 flagship models, the average accuracy based on subject was lowest for chemistry (10.3/20, 51.3%), followed by physics (15.3/20, 76.3%). All the other subjects achieved an accuracy exceeding 80%.

For questions that consisted of only text, most models exhibited high accuracy, with a few exceptions. Three models (ChatGPT o1 preview, ChatGPT o1, and Perplexity Pro) showed a correct answer rate of over 90%. The accuracy decreased greatly for questions that included diagrams; the average of all 18 models was 36.7% (22.4/61) and was 50.8% (31.0/61) when models that could not input diagrams were excluded. For questions that included figures, Claude 3.5 Sonnet (new) showed the highest accuracy (47/61, 77%). For the calculation questions, the most recent model showed an improvement in accuracy but did not achieve high accuracy for questions that included chemical structures (Table 4).

**Table 3.** Accuracy and number of correct answers according to subject for each OC-LLM^a^ in the 107th JNLEP^b^^,c^.

OC-LLM^a^	Subject^d^																	
	Physics (n=20)		Chemistry (n=20)		Biology (n=20)		Hygiene (n=40)		Pharmacology (n=40)		Pharmaceuticals (n=40)		Pathophysiology (n=40)		Regulations (n=30)		Practice (n=95)	
	Correct answers, n	Accuracy	Correct answers, n	Accuracy	Correct answers, n	Accuracy	Correct answers, n	Accuracy	Correct answers, n	Accuracy	Correct answers, n	Accuracy	Correct answers, n	Accuracy	Correct answers, n	Accuracy	Correct answers, n	Accuracy
ChatGPT																		
GPT-3.5	4	0.200	3	0.150	5	0.250	13	0.325	19	0.475	11	0.275	27	0.675	10	0.333	42	0.442
GPT-4^c^	11	0.550	7	0.350	13	0.650	28	0.700	36	0.900	25	0.625	28	0.700	20	0.667	82	0.863
GPT-4o	13	0.650	12	0.600	19	0.950	33	0.825	39	0.975	31	0.775	35	0.875	25	0.833	87	0.916
GPT-4o mini	9	0.450	2	0.100	7	0.350	23	0.575	36	0.900	18	0.450	31	0.775	21	0.700	68	0.716
o1 mini	12	0.600	5	0.250	9	0.450	26	0.650	32	0.800	25	0.625	34	0.850	16	0.533	75	0.789
o1 preview	14	0.700	6	0.300	10	0.500	27	0.675	39	0.975	28	0.700	36	0.900	24	0.800	88	0.926
o1	14	0.700	8	0.400	18	0.900	34	0.850	39	0.975	34	0.850	38	0.950	25	0.833	89	0.937
Gemini																		
1.0 Pro	6	0.300	6	0.300	12	0.600	21	0.525	22	0.550	15	0.375	24	0.600	19	0.633	46	0.484
1.5 Pro	14	0.700	4	0.200	11	0.550	25	0.625	32	0.800	25	0.625	36	0.900	23	0.767	76	0.800
1.5 Flash	11	0.550	9	0.450	17	0.850	29	0.725	32	0.800	24	0.600	32	0.800	22	0.733	66	0.695
2.0 Flash	17	0.850	11	0.550	13	0.650	35	0.875	37	0.925	33	0.825	35	0.875	27	0.900	80	0.842
Claude																		
3 Sonnet	9	0.450	7	0.350	14	0.700	30	0.750	28	0.700	3	0.075	32	0.800	18	0.600	53	0.558
3 Haiku	8	0.400	8	0.400	11	0.550	26	0.650	32	0.800	21	0.525	31	0.775	21	0.700	55	0.579
3 Opus	11	0.550	3	0.150	16	0.800	30	0.750	35	0.875	27	0.675	34	0.850	24	0.800	80	0.842
3.5 Sonnet	15	0.750	8	0.400	16	0.800	36	0.900	37	0.925	32	0.800	36	0.900	28	0.933	85	0.895
3.5 Sonnet (new)	14	0.700	10	0.500	19	0.950	35	0.875	38	0.950	33	0.825	36	0.900	25	0.833	87	0.916
Perplexity																		
Standard	11	0.550	2	0.100	6	0.300	23	0.575	38	0.950	25	0.625	34	0.850	21	0.700	68	0.716
Pro	16)	0.800	12	0.600	18	0.900	35	0.875	40	1.000	33	0.825	38	0.950	28	0.933	81	0.853

a OC-LLM: online chat-based large language model.

b JNLEP: Japanese National License Examination for Pharmacists.

c GPT-4 results were obtained from Sato and Ogasawara [14].

d The mean (SD) for physics is 0.581 (0.172), chemistry is 0.342 (0.162), biology is 0.650 (0.223), hygiene is 0.707 (0.151), pharmacology is 0.849 (0.147), pharmaceuticals is 0.615 (0.211), pathophysiology is 0.829 (0.095), regulations is 0.735 (0.15), and practice is 0.765 (0.157).

**Table 4.** Accuracy and number of correct answers based on question type for each model of OC-LLM^a^ in the 107th JNLEP^b^^c^^d^

OC-LLM^a^	Question type									
	Text (n=284)		Diagram (n=61)		Calculation (n=18)		Graph (n=16)		Chemical structure (n=19)	
	Answers, n	Accuracy	Answers, n	Accuracy	Answers, n	Accuracy	Answers, n	Accuracy	Answers, n	Accuracy
ChatGPT										
GPT-3.5	134	0.472	0	0.000	5	0.278	0	0.000	0	0.000
GPT-4^c^	227	0.799	22	0.361	9	0.500	5	0.313	4	0.211
GPT-4o	254	0.894	39	0.639	12	0.667	7	0.438	10	0.526
GPT-4o mini	215	0.757	0	0.000	8	0.444	0	0.000	0	0.000
o1 mini	231	0.813	0	0.000	15	0.833	0	0.000	0	0.000
o1 preview	271	0.954	0	0.000	15	0.833	0	0.000	0	0.000
o1	260	0.915	39	0.639	16	0.889	9	0.563	7	0.368
Gemini										
1.0 Pro	158	0.556	13	0.213	4	0.222	4	0.250	2	0.105
1.5 Pro	233	0.820	13	0.213	9	0.500	2	0.125	3	0.158
1.5 Flash	211	0.743	31	0.508	7	0.389	7	0.438	7	0.368
2.0 Flash	246	0.866	42	0.689	13	0.722	9	0.563	10	0.526
Claude										
3 Sonnet	182	0.641	28	0.459	9	0.500	9	0.563	5	0.263
3 Haiku	190	0.669	27	0.443	12	0.667	7	0.438	5	0.263
3 Opus	228	0.803	23	0.377	5	0.278	6	0.375	4	0.211
3.5 Sonnet	250	0.880	42	0.689	15	0.833	12	0.750	8	0.421
3.5 Sonnet (new)	250	0.880	47	0.770	16	0.889	11	0.688	11	0.579
Perplexity										
Standard	226	0.796	0	0.000		0.444	0	0.000	0	0.000
Pro	264	0.930	37	0.607		0.556	8	0.500	11	0.579

a OC-LLM: online chat-based large language model.

b JNLEP: Japanese National License Examination for Pharmacists.

c GPT-4 results obtained from Sato and Ogasawara [14].

d The mean (SD) for text is 0.788 (0.131), diagram is 0.367 (0.279), calculation is 0.580 (0.219), graph is 0.333 (0.257), and chemical structure is 0.254 (0.213).

### Statistical Analysis of Improved Accuracy and Response Consistency

The GLMM analysis demonstrated that the accuracy of the latest flagship models was significantly higher than that of earlier models (*P*<.001). In addition, questions containing diagrams had significantly lower accuracy than that of text-only questions (*P*<.001). The interaction term between the flagship status and question type was not significant (*P*=.53). Therefore, the difference in accuracy between the early and most recent models was consistently observed, regardless of whether the questions included diagrams or were text-based. Moreover, the flagship models did not show a greater improvement in the accuracy of diagram-based questions. The Fleiss’ κ value was 0.334, thus verifying the consistency of each question for the 4 flagship models in the 345 questions.

## Discussion

### Overview

This study evaluated the performances of 18 generative AI models in the pharmacy field by applying the same prompt to an identical input task of the 107th JNLEP. Although previous studies evaluated the performance of generative AI in the health care field using several models, this study is the first to directly compare several OC-LLMs under identical conditions for the same task. Recent meta-analyses have evaluated the performance of generative AI in health care [21,22]. However, as individual studies differ in language, prompts, and input tasks, inherent limitations exist in terms of interpreting these results.

Among these models, Perplexity Pro achieved the highest overall accuracy (301/345, 87.2%). When restricted to text-only questions, the ChatGPT o1 preview demonstrated the highest accuracy (271/284, 95.4%). For questions including diagrams, Claude 3.5 Sonnet (new) demonstrated the best performance (47/61, 77%). In early multimodal models, such as ChatGPT GPT-4 and Gemini 1.0 Pro, the accuracy for questions with diagrams was low. However, the accuracy of the latest versions of the flagship models has significantly improved. These findings indicate that the ability to recognize diagrams advanced markedly over the past year.

In terms of overall accuracy, the 4 flagship models exceeded not only the passing criteria but also the average examinee score of 68.2% [14]. This suggests that the current generative AI may possess a more extensive knowledge base than that of novice human pharmacists. However, even the best models had over 10% incorrect answers (ie, hallucinations); therefore, these models must be interpreted with caution, especially in health care.

Subject-specific analysis demonstrated accuracy improvements for all subjects when using the latest 4 flagship models. The performance for the subjects of hygiene and regulations tends to be weaker [16,23,24]. This is likely due to the influence of country-specific health care systems and social contexts, which may not be fully covered by pretraining data. In addition, the low accuracy observed in basic science subjects (physics, chemistry, and biology) is consistent with the trends reported in previous studies [25]. However, even in these subjects, improvements in accuracy were observed with the 2024 flagship models; hence, previous weaknesses may have been overcome. This improvement is likely attributable to the enhanced training data, increased model parameters, and the implementation of multimodal and reasoning capabilities. Although improvements in accuracy were observed, the flagship models still showed low accuracy in subjects, such as chemistry (10.3/20, 51.3%) and physics (15.3/20, 76.3%). This may be because these subjects included many questions that required abilities beyond factual knowledge, including calculations and image recognition. Low accuracy in chemistry has also been reported in previous studies [26].

Question type–specific analysis revealed lower accuracy for items that required image recognition or calculation, relative to text-only questions. Considering that image recognition and calculation are abilities that conventional large language models are not designed to handle and are acquired later through multimodal integration, the insufficient performance in this domain may be due to the incomplete maturation of learning.

Among the diagram-based questions, those involving chemical structures exhibited the lowest accuracy. The small mean and SD across all models for chemistry indicate that the performance of the current models did not show a considerable improvement. This may be because of two factors: (1) chemical structures are foundational scientific knowledge needed exclusively by pharmacists, leading to limited web-based availability (ie, reduced opportunities for large language model pretraining); and (2) interpreting chemical structures requires more sophisticated image recognition skills than that required for the interpretation of tables or graphs.

Claude 3.5 Sonnet (new) demonstrated the highest accuracy across all 3 types of questions—computation, graph interpretation, and chemical structure recognition. However, Claude’s flagship model showed lower accuracy for text-based questions than that of the ChatGPT and Perplexity flagship models. Therefore, a novel finding of this study is that the top-performing model differed according to the question type.

The GLMM analysis demonstrated a significant increase in overall accuracy by 2024. Although improvements in the accuracy of the questions containing diagrams were observed in the individual models, these differences were not statistically significant. The tendency for lower accuracy on diagram-based questions persisted even in the flagship models.

According to Landis and Koch [27], a Fleiss’ κ of 0.344 among the 4 flagship models indicates a certain degree of consistency. This result suggests that although these models handle simpler questions similarly, their incorrect answers differ across more challenging questions, thus indicating variations in their strengths and weaknesses. Initially, it was hypothesized that the types of questions with which each OC-LLM service struggles would differ. Correspondingly, the observation that even the flagship models with high overall accuracy failed to achieve substantial response agreement, as measured by the κ coefficient, supports this hypothesis. Therefore, identifying the specific domains in which each OC-LLM service underperforms remains an important subject for future research, including meta-analysis.

In this study, each model was evaluated using the same task to compare their performance directly. Some models included in this study have been deprecated and are no longer available. Although many new OC-LLMs are expected to emerge in the future, evaluating their performance using the 107th JNLEP will enable their comparison with previous models. Ultimately, the 107th JNLEP can serve as a benchmark for evaluating the performance of generative AI models in the field of pharmacy in Japan.

In this study, questions from the Japanese National Pharmacist Examination were input in Japanese in their original format. Translating non-English tasks into English should improve the accuracy of AI [28-31]. Therefore, this study evaluated the performance of AI models in the pharmaceutical field using Japanese input. However, higher accuracy may be achieved when questions are input using English translations. The accuracy of each model is based on the evaluation time, and the same model may show improved performance due to upgrades. Perplexity has been upgraded multiple times; however, the available models remain as Standard and Pro versions, and the version information is not disclosed to users.

Although the highest-performing model among the 18 OC-LLMs in this study achieved an accuracy of 87.2% (301/345), it also indicated that over 12.8% (n=44 questions) of the responses were incorrect (ie, hallucinations). With the improved performance of the OC-LLMs, it is anticipated that their use by medical and pharmacy students for inputting national examination questions for self-study will increase. However, as the latest models generate logical and fluent answers, it has become increasingly difficult to identify hallucinations. Even when using flagship models in 2024, the following approaches to reduce the risk of hallucinations are required in medical applications: limit use to cases in which users can independently determine the correctness of the output or confirm the supporting source information through the links provided.

The performance improvements of the OC-LLMs in this study may facilitate their broader integration into routine pharmacy practice in the near future. In clinical pharmacy practice, responding to inquiries from patients and health care professionals regarding drug information is a frequent task. These inquiries include questions about adverse drug reactions, drug interactions, dosage adjustments, or contraindications. Suitably, support from high-performance OC-LLMs is expected to improve the quality of responses and reduce the time required to address such inquiries. The use of OC-LLMs in direct medical support, for example, in selecting personalized pharmacological treatments, requires careful consideration of ethical issues, such as explainability, responsibility, privacy, and patient rights.

### Principal Findings

This study evaluated the performance of 18 OC-LLMs available in 2024, based on questions from the National Pharmacist’s License Examination in Japan. As the models were upgraded, their accuracy improved. The performance of the flagship models exceeded both the passing criteria and the examinees’ average score. In the latest versions of the OC-LLMs, enhancements in multimodal capabilities significantly improved accuracy in both interpreting charts and figures and solving calculation-based questions. Furthermore, the answer consistency of the flagship models was not robust, which suggests that each model had different strengths and weaknesses.

### Limitations

In this study, only a single set of examination questions was tested, and each question was entered only once. Generative AI has a characteristic known as temperature, which refers to the inherent variability in its responses. This means that the model can generate different answers even when given the same question. Therefore, if the test is repeated, the accuracy of each OC-LLM method can vary. Several studies have evaluated OC-LLM performance by testing questions over multiple years [32-34] or conducting multiple rounds of testing [35]. However, similar to many previous studies, to evaluate the 18 models within a limited time frame, only 1 set of questions was administered per examination year to each model. Human examinees also underwent the national pharmacist’s examination only once, rendering the testing conditions comparable. The 107th JNLEP comprises 345 questions, with multiple items allocated to each subject and question type. Therefore, the examination is considered sufficient to allow for a certain degree of interpretation.

With the progressive improvement of the models over time, the top-performing service shifted from ChatGPT to Claude, and subsequently to Gemini. Across OC-LLM services, such as ChatGPT, Gemini, Claude, and Perplexity, no consistent patterns were observed across subjects or question types. Considering that the key information, such as the volume of pretraining data, number of parameters, and tuning strategies of these OC-LLMs, is not publicly disclosed, fully discussing the factors that contribute to their improved performance in the pharmaceutical field is difficult. These factors include understanding of diagrams, chemical structures, and calculation-based questions.

### Comparison With Prior Work

Numerous studies have evaluated the performance of OC-LLM in terms of knowledge of health care license examinations (Table 5). Early OC-LLMs failed the National Medical License Examination; however, the subsequent release of high-performance OC-LLMs met the passing criteria.

The reported OC-LLMs in Table 5 are biased toward ChatGPT, and the challenges and conditions vary according to each report on medical performance. Moreover, the performance of OC-LLM declines in languages other than English because of the smaller volume of training data [18,25,34-36]. Therefore, verifying the performance of OC-LLM in non-English languages is important. An important contribution of this study is its demonstration that multiple flagship OC-LLMs substantially outperform the passing criteria in areas where prior evidence is scarce, specifically in non-English languages and the pharmaceutical field. Achieving high response accuracy from OC-LLMs using non-English prompts has considerable implications for clinical implementation in health care settings in Japan (and other non-English–speaking regions).

One of the major strengths of this study is its systematic evaluation of multiple OC-LLMs released in 2024 under identical input conditions, such as the same prompt text and image resolution or size. To the best of our knowledge, this is the first study to evaluate the longitudinal improvement in generative AI performance in medical examinations.

**Table 5.** Studies evaluating the performance of generative AI^a^ in health care licensing examinations.

Health care license examination study	Country or region	OC-LLM^b^	Accuracy (%)
Medical license			
Gilson et al (2023) [36]	United States	GPT-3	25.3
Gilson et al (2023) [36]	United States	ChatGPT (unknown)	64.4, 57.8
Flores-Cohaila et al (2023) [37]	Peru	ChatGPT GPT-3.5	77
Flores-Cohaila et al (2023) [37]	Peru	ChatGPT GPT-4	86
Jung et al (2023) [38]	Germany	ChatGPT (unknown)	60.1, 66.7
Shang et al, (2023) [28]	China	ChatGPT GPT-3.5	57
Wang et al, (2023) [39]	China	ChatGPT GPT-3.5	56
Wang et al, (2023) [39]	China	ChatGPT GPT-4	84
Yanagita et al (2023) [40]	Japan	ChatGPT GPT-3.5	42.8
Yanagita et al (2023) [40]	Japan	ChatGPT GPT-4	81.5
Takagi et al (2023) [41]	Japan	ChatGPT GPT-3.5	50.8
Takagi et al (2023) [41]	Japan	ChatGPT GPT-4	79.9
Tanaka et al (2024) [16]	Japan	ChatGPT GPT-3.5	52.9, 63.6
Tanaka et al (2024) [16]	Japan	ChatGPT GPT-4	85.6
Liu et al (2025) [42]	Japan	ChatGPT GPT-4	77
Liu et al (2025) [42]	Japan	ChatGPT GPT-4o	89
Liu et al (2025) [42]	Japan	Gemini 1.5 Pro	80
Liu et al (2025) [42]	Japan	Claude 3 Opus	82
Oztermeli and Oztermeli (2023) [43]	Turkey	ChatGPT GPT-3.5	64.7, 67.1, 70.9, 60.8, 54.3
Siebielec et al (2024) [32]	Poland	ChatGPT GPT-3.5	59.5, 57.5, 63.5, 62.0, 61.0
Wójcik et al (2024) [31]	Poland	ChatGPT GPT-4	67.1
Pharmacist license			
Wang et al (2023) [44]	Taiwan	ChatGPT (unknown)	54.5, 63.5
Wang et al (2025) [45]	Taiwan	ChatGPT GPT-3.5	59
Wang et al (2025) [45]	Taiwan	ChatGPT GPT-4	73
Kunitsu (2023) [46]	Japan	ChatGPT GPT-4	78.2, 75.3
Sato and Ogasawara (2024) [14]	Japan	ChatGPT GPT-3.5	45.5
Sato and Ogasawara (2024) [14]	Japan	ChatGPT GPT-4	72.5
Jin and Kim (2024) [47]	Korea	ChatGPT GPT-3.5	61
Jin and Kim (2024) [47]	Korea	ChatGPT GPT-4	87
Nurse license			
Taira et al (2023) [33]	Japan	ChatGPT GPT-3.5	71, 71, 63, 63, 63
Kaneda et al (2023) [48]	Japan	ChatGPT GPT-3.5	59.9
Kaneda et al (2023) [48]	Japan	ChatGPT GPT-4	79.7
Wu et al (2024) [49]	China	ChatGPT GPT-3.5	51.7
Wu et al (2024) [49]	China	ChatGPT GPT-4	70.5
Wu et al (2024) [49]	China	Google Bard	48.3
Hiwa et al (2024) [50]	Unknown	ChatGPT GPT-3.5	77
Hiwa et al (2024) [50]	Unknown	Gemini (unknown)	75
Hiwa et al (2024) [50]	Unknown	Microsoft Copilot	84
Hiwa et al (2024) [50]	Unknown	Llama2	68

a AI: artificial intelligence.

b OC-LLM: online chat-based large language model.

### Conclusions

This study reveals that the performance of OC-LLMs in the pharmaceutical field has greatly improved as of 2024. Particularly, an increase in accuracy was observed for questions with diagrams. In the most recent version of the models, evaluated in 2024, the overall accuracy reached 85%, markedly exceeding the average examinee score, and indicating their potential as valuable support tools. Although caution is necessary due to the potentially serious impact of hallucinations on health care, the benefits of OC-LLMs outweigh the associated risks. Accordingly, health care professionals and medical educators must acquire the skills necessary to effectively use OC-LLMs, particularly the ability to recognize and manage hallucinations.

## Supplementary material

10.2196/76925 Multimedia Appendix 1 The responses of all the online chat-based large language models to each question.

## Abbreviations

AI: artificial intelligence
GLMM: generalized linear mixed model
JNLEP: Japanese National License Examination for Pharmacists
OC-LLM: online chat-based large language model
